# Case report: Overlapping syndrome of MOG-IgG associated optic neuritis and autoimmune encephalitis with co-existence of anti-NMDAR and anti-GABA_B_R antibodies

**DOI:** 10.3389/fimmu.2024.1461024

**Published:** 2025-01-14

**Authors:** Zhujun Mei, Jingguo Wang, Junling Wang, Xiaoni Liu, Bo Deng, Wenbo Yang, Xiang Zhang, Xiangjun Chen, Hai Yu

**Affiliations:** ^1^ Department of Neurology, Huashan Hospital, Fudan University and Institute of Neurology, Fudan University, Shanghai, China; ^2^ Department of Neurology, National Center for Neurological Disorders, Shanghai, China; ^3^ Department of Neurology, Xiangya Hospital, Central South University, Jiangxi (National Regional Center for Neurological Diseases), Nanchang, Jiangxi, China; ^4^ Jiangxi Provincial People’s Hospital, Clinical College of Nanchang Medical College, First Affiliated Hospital of Nanchang Medical College, Nanchang, Jiangxi, China

**Keywords:** overlapping syndrome, autoimmune encephalitis (AE), N-methyl-D-aspartate receptor (NMDAR), myelin oligodendrocyte glycoprotein (MOG), gamma-aminobutyric acid-B receptor (GABA_B_R), optic neuritis (ON)

## Abstract

We report a case of optic neuritis (ON) secondary to autoimmune encephalitis (AE) in a patient with concomitant antibodies to N-methyl-D-aspartate receptor (NMDAR), gamma-aminobutyric acid-B receptor (GABA_B_R), and myelin oligodendrocyte glycoprotein (MOG). The patient exhibited a constellation of symptoms, including vision loss, seizures, mental and behavioral disorders, cognitive impairment, and speech abnormalities. At the two-year follow-up, the patient’s symptoms had abated entirely. Overlap syndrome of triple autoimmune antibodies is rare and the coexistence of antibodies to NMDAR, GABA_B_R and MOG has not been reported till now. This case report provides novel experience of diagnosis and treatment in autoimmune overlap syndromes.

## Introduction

With the continued discovery of novel autoimmune antibodies and the expansion of the clinical syndrome spectrum, there is a notable rise in the number of cases of AE with multiple antibody positivity. Comprehensive analyses of patients with concurrent or sequential anti-NMDAR and demyelinating antibodies (e.g. anti-MOG IgG and anti-aquaporin 4 (AQP4) IgG) have been performed (1). However, the incidence of cases where multiple neuronal and demyelinating antibodies have been identified in the same subject remains low. In this study, we present a case with the simultaneous presence of anti-MOG, anti-NMDAR, and anti-GABA_B_R. with the aim of expanding our view of AE in the presence of multiple antibodies. This study contributes to expanding our understanding of AEs in which multiple antibodies are present, and the concept of “culprit antibodies” (2).

## Case report

This study was approved by the Ethics Committee of Huashan Hospital, Fudan University. The patient provided written informed consent and agreed to sample collection and data publication in this article.

The serum samples from the first hospitalization were tested at Hangzhou Dunen Medical Laboratory. The cerebrospinal fluid (CSF) and serum samples from the second and third hospitalization were sent to Jiangsu Xiansheng Inspection Company (Nanjing, China) for testing.

**Figure 1:**
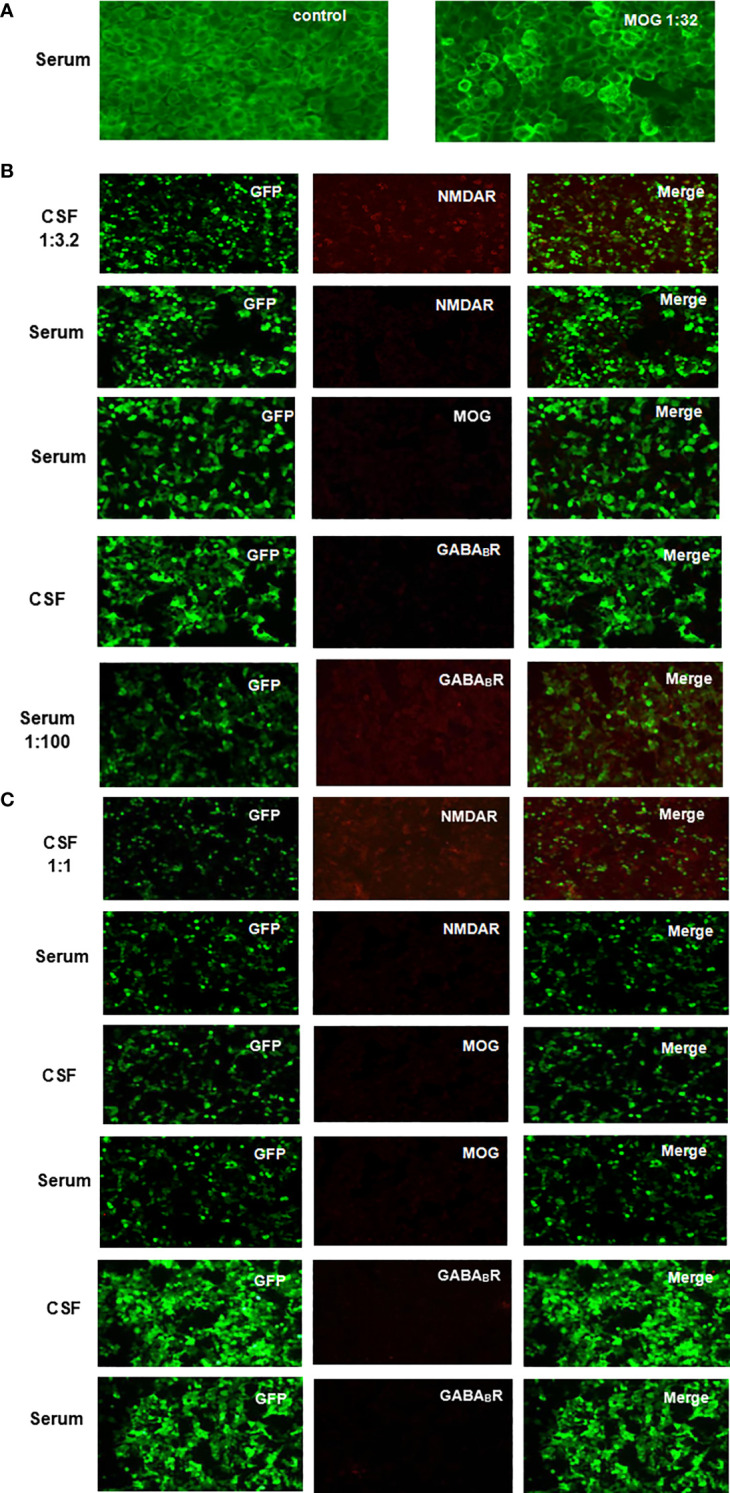
Anti-NMDAR, MOG and GABA_B_R IgG were detected in patient CSF and serum by indirect immunofluorescence on CBA using unfixed HEK293 cells transfected with plasmids. **(A)** Fluorescent antibody staining to detect anti-MOG IgG expression in serum after the onset of vision loss. **(B)** Fluorescent antibody staining of serum anti-MOG IgG and CSF and serum anti-NMDAR and anti-GABA_B_R IgG expression was performed 4 months after recurrent seizures with a range of psychiatric, behavioral cognitive, and language disorder symptoms. **(C)** Fluorescent antibody staining was used to detect the expression of anti-MOG, anti-NMDAR, and anti-GABA_B_R IgG in CSF and serum at 2 weeks after first-line immunotherapy.

**Figure 2:**
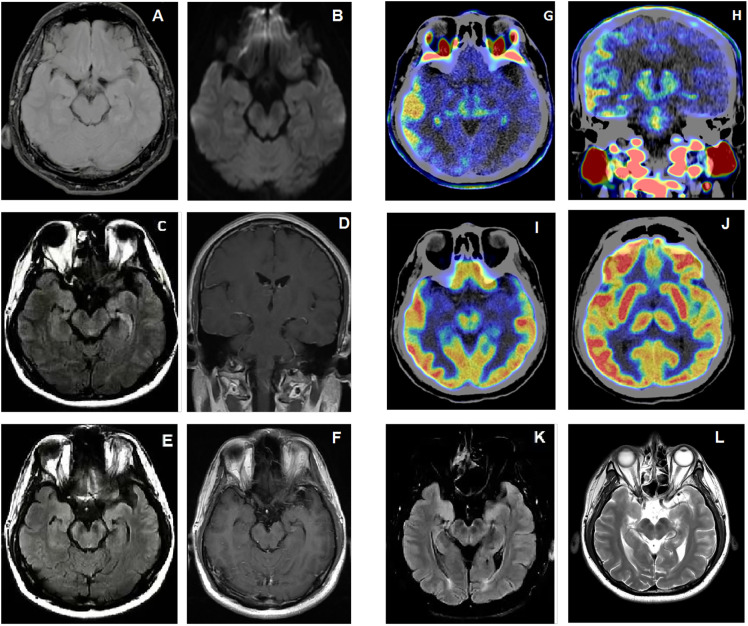
**(A, B)** On the first admission, FLAIR and DWI sequences showed normal **(C, D)** On the second admission, FLAIR sequences and Contrast-Enhanced T1-weighted sequences of the patient with a series new symptoms onset revealed swelling temporal lobe and enhanced leptomeningeal of right cerebral hemisphere. **(E, F)** After the treatment of IVMP and IVIG, FLAIR sequences showed increased intensity in the bilateral hippocampi and mesencephalon, and contrast-enhanced T1-weighted sequences showed patchy linear enhancement in the meninges. **(G, H)** 18F-DPA714 PET/CT showed higher distribution of 18F-DPA714 in right frontal temporoparietal lobe. **(I, J)** 18F-FDG PET/CT showed hyper metabolism of fluorodeoxyglucose in right frontal temporoparietal lobe. **(K, L)** After 1 year follow up, FLAIR and T2-weighted sequences showed reduced lesions in the bilateral hippocampi and mesencephalon compared to before.

In February 2021, a previously healthy 31-year-old male patient presented with acute vision loss and eye pain for a one-week period. Ophthalmologic examination revealed hand motion visual acuity and optic disc edema in the left eye. Serum central nervous system demyelinating antibodies were tested, which revealing positive anti-MOG IgG [titer 1:32, cell-based assay, (CBA)], (**Figure 1A**) and negative AQP4-IgG. Brain magnetic resonance imaging (MRI) did not identify any abnormalities (**Figures 2A, B**), and visual evoked potentials (VEP) demonstrated an absence of p100 amplitude in the left optic nerve. His vision loss partially resolved after he was treated with intravenous methylprednisolone (IVMP) at the local hospital (500 mg/day for 5 days; 250 mg/day for 3 days, followed by oral MP 56 mg/day, tapered by 4 mg per week).

Between July and November 2021, the patient experienced four episodes of generalized tonic-clonic seizures (GTCS) with a range of symptoms including recent memory loss, irritability, hallucinations, confusion, abnormal speech and behavior. He was readmitted to hospital on 11 November 2021 due to symptom exacerbation. Reexamination of CSF and serum autoantibodies was done with serum anti-GABA_B_ IgG (titer 1:100, CBA) and CSF anti-NMDAR IgG ((titer 1:3.2, CBA) positive. But this time serum anti-MOG IgG was negative (**Figure 1B**). Caspr2, AMPAR1, AMPAR2, LGI1, LON5, DPPX, GAD65, mGLU5, GlyR, D2R and AQP4 autoantibodies were all negative. General and enhanced MRI scan of brain revealed leptomeningeal enhancement and temporal swelling in the right cerebral hemisphere, (**Figures 2C, D**). Electroencephalogram (EEG) showed diffuse sharp and slow wave activity in right temporal lobe. VEP showed decreased amplitude of P100 in the left. For treatment, intravenous immunoglobulin (IVIG) (400 mg/kg/day for 5 days) was given along with a second wave of IVMP (500 mg/day for 5 days and 250 mg/day for 4 days) and three antiepileptic drugs (AEDs) including levetiracetam, oxcarbazepine, and sodium valproate were administered for seizure control.

Although the epilepsy had gradually been controlled, some other symptoms of recent memory loss, abnormal mental and behavior remained. Therefore, the patient was referred to Hushan hospital for further treatment. Neurological examination on admission showed he had cognitive dysfunction. Mini-mental State Examination score (MMSE) was 20. Lumbar puncture showed normal pressure (160 mmH_2_O), CSF protein level (367 mg/L, normal range < 400 mg/L) and cell count (leucocytes, 1 x 10^6^/L, 91% mononuclear, red blood cell 0x 10^6^/L). The CSF oligoclonal IgG bands were negative. CBA revealed positive anti-NMDAR in CSF (titer 1:1) without any other overlapping antibodies (**Figure 1C**). Positive anti-thyroid peroxidase (102.0U/ml, normal range < 35 U/ml), anti-thyroglobulin (438.0U/ml, normal range < 115 U/ml) and anti-Ro-52 antibodies were detected. Labial gland biopsy was negative. Ultrasound showed bilateral TI-RADS3 thyroid nodules without thyroid enlargement. Serological neoplastic parameters (AFP, CEA, CA199, CA125, NSE, SCCA, CYFRA21-1, t-PSA, f-PSA) and whole-body CT showed no evidence of neoplastic disease. Repeated brain MRI with contrast revealed hyperintensity of bilateral hippocampi and mesencephalon on FLAIR sequences And patchy linear enhancement of leptomeninges on contrast-enhanced T1-weighted sequences (**Figures 2E, F**). EEG showed scattered and paroxysmal 4-7 Hz θ waves. Optical coherence tomography (OCT) showed no abnormality and the left visual acuity returned to normal. He was diagnosed with anti-MOG related ON and AE overlap syndrome with anti-NMDAR and anti-GABA_B_ IgG, and continued to be treated with IVMP (120mg/day for 5days, 80mg/day for 4 days) and oral prednisone (60mg/day; followed by 5mg dose reductions every fortnight). The symptoms improved as MP dose decreased. Meanwhile, ^18^F-FDG PET/CT and ^18^F-DPA714 PET/CT showed hyper metabolism of glucose and inflammatory infiltrate in the right frontotemporal parietal lobe (**Figures 2G–J**). Then the rituximab (RTX) was administered (100mg 1^st^ day, 500mg 2^nd^ day) to prevent relapse. Adverse effect wasn’t observed during the administration of MP and RTX. At discharge, his symptoms were nearly in complete remission, and MMSE improved to 27/30.

**Figure 3:**
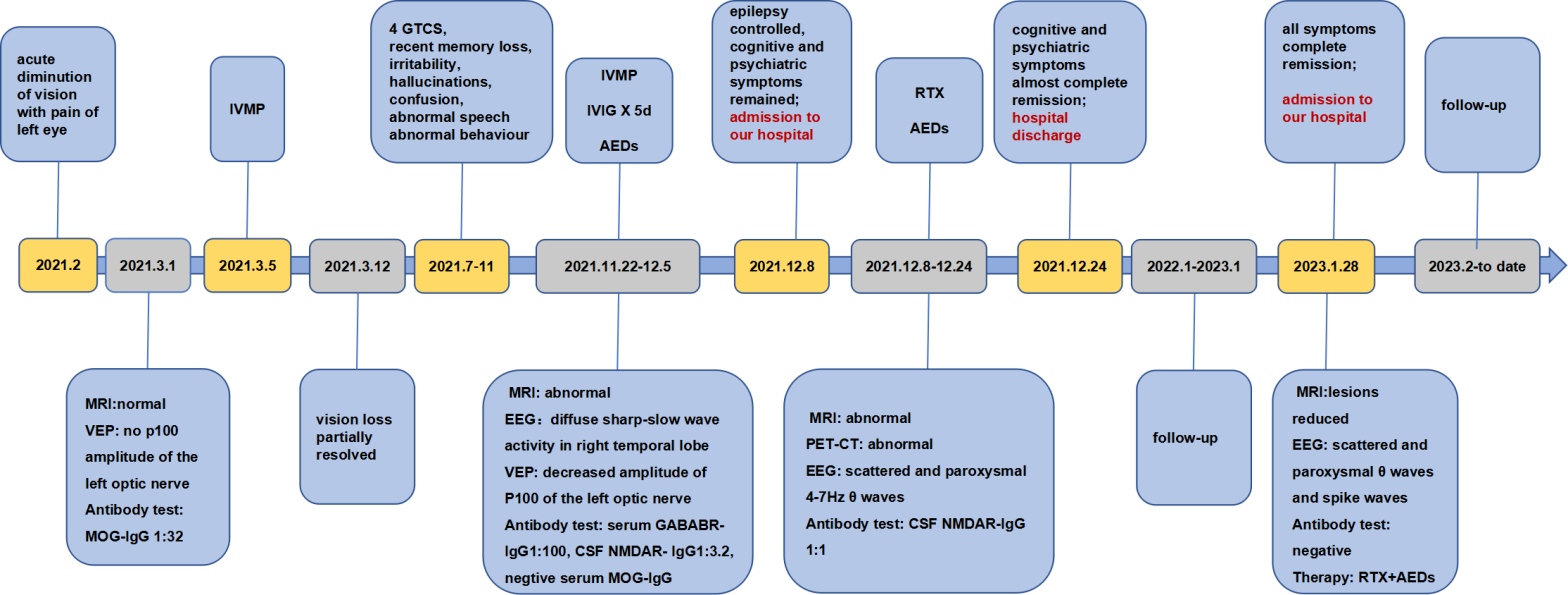
Timeline of the development of symptoms, key findings from imaging and laboratory tests, diagnosis and treatment.

The patient was well followed every 3 months from January to December 2023. The autoantibody test revealed consistent negative anti-NMDAR IgG, while the anti-GABAB IgG titers show variability, ranging from negative to weakly positive. He remained seizure-free and stable post-discharge. In January 2023, he was admitted to the hospital for a comprehensive examination. Visual acuity, OCT, CSF analysis, and autoantibody tests all came back normal. Tumor screening was negative. MRI plain scan of brain showed significant reduction in bilateral hippocampal and mesencephalic lesions (**Figures 2K, L**). The EEG showed scattered and paroxysmal θ waves and spike waves. He continued AEDs and RTX. B lymphocyte subsets were detected to assess the effectiveness and tolerability of RTX. Follow-up indicated stable condition. Good adherence of RTX maintained due to effectiveness of treatment and no infection event. The clinical course for this patient was summarized in **Figure 3**.

## Discussion

Increasing studies have focused on neuronal or glial cell antibodies and antigens. Anti-MOG IgG may act on MOG on the surface of oligodendrocytes to mediate demyelination and damage myelin sheaths through antibody-dependent cellular cytotoxicity or complement activation (3). Anti-NMDAR binds to the GluN1 subunit of NMDAR, altering its surface dynamics and interactions with other synaptic proteins, internalizing it and leading to a reduction in NMDAR (4). GABA_B_R, which is mainly distributed in the cerebral cortex, hippocampus, cerebellum, and thalamus, is an inhibitory synaptic protein in neurons, and plays an important role in in the transmission of neurotransmitters and synaptic plasticity (5).

Approximately 4% to 7.5% of patients with anti-NMDAR encephalitis will also develop glial cell antibodies or neuronal surface antibodies. The most common association is that of anti-NMDAR with anti-MOG or AQP4 antibodies, which can lead to overlapping symptoms (6). One study collected 846 anti-NMDAR encephalitis patients and showed that 42 patients had at least one additional neuronal surface antibodies or neuroglial antibodies: 17 of 30 patients with neuroglial antibodies had anti-MOG IgG and 1 of 12 patients with neuronal surface antibodies had anti-GABA_B_R IgG (6). Although the coexistence of triple or more autoimmune antibodies is rare, there are some reports about the overlap syndrome. A study from Cleveland Clinic containing 42,032 patients tested for AE-Abs in serum or CSF showed 2 patients with triple AE-Abs and 1 patients with 4 AE-Abs (7). A case of coexistent NMDAR, CASPR2 and MOG antibodies was reported by Cherian A, et al. as well (8). The mechanism of antibody coexistence is not well understood, and the concept of epitope spreading may partially explain this phenomenon, whereby sustained recognition and activation of self-antigens induces a chronic immune response with the simultaneous production of antibodies directed against different dominant epitopes in the same antigen or against different antigens (9). Another view is that oligodendrocyte damage associated with MOGAD may lead to secondary damage to NMDAR, due to the co-existence of MOG and NMDAR on oligodendrocytes (1). Coexistence of multiple autoimmune antibodies could be explained by general susceptibility for autoimmune processes as well. It may be a consequence of immune dysregulation. Genetic predisposition and an environmental trigger factor may also be the mechanism of overlapping syndrome (10). Autoimmune encephalitis often associates with systemic autoimmune diseases, malignancies, infectious diseases. 3 other autoimmune antibodies have been detected in the patient of our case, but the results didn’t reach the diagnostic standards of autoimmune disease. Furthermore, no malignancy was found this time. Ongoing cancer screenings is necessary after diagnosis.

In this case, the diagnosis of MOG-IgG associated ON was definite according to the symptom of visual loss, the positive serum anti-MOG IgG and the improved symptom after administration of IVMP, although the absence of p100 amplitude has not been reported in MOGAD-ON before. According to the other published articles, the presence of optic disc swelling in acute phase and better clinical recovery meet the clinical features of MOGAD-ON (11). However, a series of new symptoms followed, suggesting possible cortical damage. Subsequently, anti-GABA_B_ IgG was detected in serum and anti-NMDAR IgG in CSF. It is critical to identify the culprit antibody in this patient. Previous studies have suggested several key points for the culprit antibody. Firstly, the titer of antibodies should correlate with the disease severity, treatment response and prognosis. Secondly, antibodies should have a clear causal relationship with a specific clinical phenotype (2). In terms of this patient, the titers of all antibodies decreased and symptoms gradually resolved after treatment with first-line immunotherapy. With second-line immunotherapy, symptoms almost completely disappeared and all antibodies turned negative. This suggested that the titers of the three antibodies correlate with the severity of the disease and the response to treatment.

The most frequent symptom of anti-GABA_B_R encephalitis is refractory seizure, and GTCS is the most common seizure type (12). The patient of our case experienced multiple GTCS. This is consistent with anti-GABA_B_R encephalitis. However, seizures are also common in anti-NMDAR encephalitis. A study contained 153 patients with AE, 57% of 75 patients with anti-NMDAR encephalitis and 84% of 25 patients with anti-GABA_B_R encephalitis had seizures (13). Seizures, cognitive deficits, speech abnormalities, and mental and behavioral disorders are common symptoms of both anti-NMDAR encephalitis and anti-GABA_B_R encephalitis. Therefore, we believe that these symptoms may be mediated by either anti-NMDAR or anti-GABA_B_R IgG. However, the effect of each antibody on these symptoms is also speculative. A systematic review containing 114 anti-GAGA_B_R encephalitis revealed the medial temporal lobe (MTL) and hippocampus were most common lesion sites in MRI. Lesions on frontal, temporal, and parietal lobe also have been reported (12). The most common lesion includes T2 hyperintensities in MTL of anti-NMDAR encephalitis (8). Lesions involving bilateral MTL and hippocampus are common to both anti-GABA_B_R and anti-NMDAR encephalitis.

In this case, ^18^F-FDG and ^18^F-DPA714 PET-CT was performed as a diagnostic tool. A study of anti-NMDAR encephalitis showed ^18^F-FDG reflecting metabolic pattern could correlate with the clinical course and antibody level (14). It revealed that hypermetabolism of the frontal, temporal, and parietal lobes during the acute and subacute phase, with these images turning normal as symptoms recovered (14).^18^F-DPA714 reflects the distribution of translocator protein 18kDa (TSPO), which is expressed predominantly in activated microglia and is a feature of neuroinflammation (15). Hyperintensity of ^18^F-FDG and ^18^F-DPA714 indicated that the patient of our case was still at inflammatory reaction, although he was treated with first-line immunotherapy. Patients with overlapping syndrome maybe at relatively higher risk of relapse, and second-line therapy can be administered to prevent relapses (16). Taken together, we decided to give RTX to reduce the risk of relapse. The patient’s condition remained stable during the two-year follow-up.

## Conclusion

We reported a rare case of concomitant of anti-MOG, anti-NMDAR and anti-GABA_B_R IgG related overlap syndrome, which enriches the spectrum of multiple-antibody-positive AE disorders known to us to date. Early diagnosis and effective treatment strategies are important for patients with multiple antibodies. Understanding culprit antibodies is necessary to understand clinical phenotypes and select accurate antibody detection. In addition, FDG-PET and TSPO-PET have recently been used as adjunctive diagnostics. Second-line immunotherapy may be necessary to reduce the recurrence of overlap syndromes. But the resources on triple autoantibodies related overlap syndrome are limited. The follow-up duration is insufficient to fully reflect all clinical features and outcomes adequately. More clinical data collection is necessary.

## Patient perspective

At the first hospitalization, I thought the disease had been controlled after the IVMP treatment. However, when I suffered from frequent seizures and couldn’t remember what had happened recently, couldn’t express myself fluently and couldn’t control my temper and behavior, I became frightened. I thank my doctors for the accurate diagnosis and effective treatment. My symptoms improved almost completely when I was discharged from Huashan Hospital Fudan University. My condition has remained stable until now, and I am full of confidence in overcoming the disease.

## Data Availability

The datasets presented in this article are not readily available because of ethical and privacy restrictions. Requests to access the datasets should be directed to the corresponding author.
