# Supplementary material for: Case report: Overlapping syndrome of MOG-IgG associated optic neuritis and autoimmune encephalitis with co-existence of anti-NMDAR and anti-GABABR antibodies
Source: Front Immunol. 2025 Jan 14;15:1461024. doi: 10.3389/fimmu.2024.1461024 (PMC11772160; doi:10.3389/fimmu.2024.1461024)
Supplement: Supplementary file 1 [file Table1.pdf]

CARE Checklist of information to include when writing a case report

CARE case report guidelines

(cc) BY-NC-ND

Topic	Item	Checklist item description	Reported on Line
Title	1	The diagnosis or intervention of primary focus followed by the words "case report"	line 1-2 on page 1
Key Words	2	2 to 5 key words that identify diagnoses or interventions in this case report, including "case report"	line 13-15 on page 2
Abstract (no references)	3a	Introduction: What is unique about this case and what does it add to the scientific literature?	line 8 -10 on page 2
	3b	Main symptoms and/or important clinical findings	line 5-7 on page 2
	3c	The main diagnoses, therapeutic interventions, and outcomes	line1-3 and 7-8 page2
	3d	Conclusion—What is the main "take-away" lesson(s) from this case?	line10-11 on page 2
Introduction	4	One or two paragraphs summarizing why this case is unique (**may include references**)	line1-12 on page 3
Patient Information	5a	De-identified patient specific information.	line 21 on page 3
	5b	Primary concerns and symptoms of the patient.	line 21-22 on page 3, line 3 - 5 on page 4
	5c	Medical, family, and psycho-social history including relevant genetic information	line 21 on page 3
	5d	Relevant past interventions withoutcomes	NA
Clinical Findings	6	Describe significant physical examination (PE) and important clinical findings.	line 21-23 on page 3, line 3-5 and 19-23 on page 4
Timeline	7	Historical and current information from this episode of care organized as a timeline	page 12
Diagnostic Assessment	8a	Diagnostic testing (such as PE, laboratory testing, imaging, surveys).	**line 23-28 on page 3,line 7-14 and 23-30 on page 4, line 1-7 on page 5**
	8b	Diagnostic challenges (such as access to testing, financial, or cultural)	NA
	8c	Diagnosis (including other diagnoses considered)	line 8-9 on page 5
	8d	Prognosis (such as staging in oncology) where applicable	line 15-16 on page 5
Therapeutic Intervention	9a	Types of therapeutic intervention (such as pharmacologic, surgical, preventive, self-care)	**line 29 on page 3,line1 and15-18 on page 4,line 9-10 and 14 on page 5**
	9b	Administration of therapeutic intervention (such as dosage, strength, duration)	**line 29 on page 3,line1 and15-18 on page 4,line 9-10 and 14 on page 5**
	9c	Changes in therapeutic intervention (with rationale)	line 14 on page 5
Follow-up and Outcomes	10a	Clinician and patient-assessed outcomes (if available)	NA
	10b	Important follow-up diagnostic and other test results	line 18-24 on page 5
	10c	Intervention adherence and tolerability (How was this assessed?)	line 26-29 on page 5
	10d	Adverse and unanticipated events	line 15 on page 5
Discussion	11a	A scientific discussion of the strengths AND limitations associated with this case report	**line 27-30 on page 8, line 1-6 on page 9**
	11b	Discussion of the relevant medical literature **with references**.	page 6-8
	11c	The scientific rationale for any conclusions (including assessment of possible causes)	page 6-8
	11d	The primary "take-away" lessons of this case report (without references) in a one paragraph conclusion	**line 27-30 on page 8, line 1-6 on page 9**
Patient Perspective	12	The patient should share their perspective in one to two paragraphs on the treatment(s) they received	line 9-15 on page 9
Informed Consent	13	Did the patient give informed consent? Please provide if requested	Yes ☑ No ☐
